# How does the content of nutrients in soil affect the health status of trees in city parks?

**DOI:** 10.1371/journal.pone.0221514

**Published:** 2019-09-11

**Authors:** Tomasz Kleiber, Michał Krzyżaniak, Dariusz Świerk, Anna Haenel, Sylwia Gałecka

**Affiliations:** 1 Department of Plant Nutrition, Poznan University of Life Sciences, Poznań, Poland; 2 Department of Landscape Architecture, Poznan University of Life Sciences, Poznań, Poland; Universita degli Studi di Perugia, ITALY

## Abstract

Trees have multi-aspect influence on the microclimate in urbanised areas. Therefore, it is important to investigate the biotic and abiotic factors affecting their health. The aim of the conducted study was to assess the chemical composition of soils and the nutritional status of lime and horse chestnut trees in selected sites and the influence of these factors on the condition and health of these tree species in urbanised areas. The research was conducted on selected trees (n = 643) growing in different parts of the city. The soils and plants were analysed for the content of macro- and microelements, sodium and heavy metals. A canonical variation analysis (CVA)–the canonical variant of Fisher's linear discriminant analysis (LDA) was used to construct the model. The CVA enabled the creation of 4 CCA models. The research showed that in general, the soil in all the sites of lime and horse chestnut trees was alkalised–at the same time it was characterised by low salinity. Despite the alkaline soil the statistical analysis showed a positive correlation between the content of manganese in the lime leaves and the deterioration of their health. In spite of that due to the satisfactory health status and condition of trees in most locations temporary guide values of nutrients were proposed for trees growing in urbanised areas. The following temporary guide values of nutrients were proposed for the horse chestnut trees (% d. m.): N 2.38%-4.71%, P 0.24%-0.46%, K 1.13%-2.31%, Ca 1.05%-2.12%, Mg 0.16%-0.42%, S 0.12%-0.23%; Fe 89.8–198.8, Zn 17.6–33.1, Cu 7.36–19.61 (mg kg^-1^ d. m.). The following temporary guide values were proposed for the small-leaved lime-trees (% d. m.): N 2.45%-3.22%, P 0.27%-0.42%, K 1.52%-2.86%, Ca 1.43%-2.02%, Mg 0.19%-0.35%, S 0.19%-0.25%; Fe 137.6–174.3, Zn 20.2–23.8, Cu 8.36–9.79 (mg kg^-1^ d. m.).

## Introduction

Trees in urbanised areas have positive influence on various aspects of city life. They help to reduce the effect of urban heat islands. They shade buildings and thus reduce the demand for energy consumed by air-conditioning systems and they improve rainwater retention [1–3]. It is also important that trees, which are part of recreational areas, have positive effect on urban residents’ health and well-being [4–8]. Trees also reduce the noise level and they purify the air from particulate matter (PM), CO_2_, O_3_ and other pollutants contained in car fumes, transport dust and generated by the industry [9–11]. Research conducted in different American cities showed that trees growing in urbanised areas could reduce air pollution with ozone, particulate matter, sulphur dioxide, carbon monoxide and nitrogen oxides [12, 13].

Trees growing in public green space are exposed to various environmental factors, damage and destruction caused by air pollution, human activity and adverse weather conditions [14–17]. As far as tolerance to urban conditions is concerned, coniferous trees are less tolerant to pollution than deciduous ones–hence the latter are more common in urbanised areas [17]. Deciduous trees are more sensitive to the exposure and effect of O_3_, whereas coniferous trees are more sensitive to the high concentration of SO_2_ and NO_2_ in the air [13]. The use of NaCl to deice roads and pavements also has harmful effect on the health of adjacent plants. Excessive salinity and exposure of plants to these conditions is manifested by adverse visual symptoms such as the loss of the natural colour of leaves and leaf necrosis and sometimes by the withering of shoots [18–21]. These conditions induce the physiological drought stress. The capture of salt aerosol (NaCl) varies depending on the tree species and leaf size. Larger *T*. *cordata* Mill. leaves adsorb more Na^+^ ions than plants with smaller leaves, e.g. *Olea* sp. Lime-trees adsorb Na^+^ ions per leaf area more effectively (0.015 mg cm^-2^) than *Quercus cerris* L. (0.009 mg cm^-2^) and *Platanus* x *hispanica* Münchh. (0.008 mg cm^-2^). Research conducted in Riga confirmed the toxic effect of NaCl on *Tilia* sp. and *A*. *hippocastanum* L. [5, 22]. Elevated concentrations of alkaline ions (such as Na, Ca, Mg) and some other ions (Cl, Zn and Cu) as well as simultaneous unfavourable increase in the soil pH adversely affect the health of *T*. *cordata* Mill. trees growing near roads. Elevated salinity (EC) caused by increased Na and Cl content has particularly unfavourable influence on the health of lime and horse chestnut trees. It causes physiological drought, especially in spring [23, 24].

Apart from that, excessive car traffic is a problem in cities. Engine exhaust gases are a significant source of heavy metals emitted in particulate matter and molecules of polyunsaturated aromatic hydrocarbons. Non-exhaust gas road emissions (wear of tyres, brake pads and discs, road surfaces and corrosion) are the source of about 50% of particulate matter PM_10_ and about 22% of PM_2.5_. To conclude, even without exhaust gas emissions car traffic is still a significant source of particulate matter (PM) in urbanised areas [25, 26]. The accumulation of heavy metals in soils and plants growing in cities has been studied in various regions of the world [27–29]. This type of pollution of anthropogenic origin is usually caused by the emission of exhaust gases and by the industry. Plants need trace amounts of some heavy metals (e.g. Fe, Mn, Zn) for proper development, because they are micronutrients from the physiological point of view. However, excessive concentrations of the same elements may have phytotoxic effect [30–33]. According to urban gradient hypothesis, which is chiefly based on research conducted in American cities, the contamination with heavy metals should decrease with distance from the city centre according to the following order: centre–urbanised city areas–urbanised suburban areas–agricultural areas–natural areas [34].

The aim of the study was to assess the impact of anthropogenic pressure on the chemical composition of soils and leaves of lime and horse chestnut trees in selected sites in Poznań, and to assess their influence on the health of trees and their tolerance to adverse habitat conditions in urbanised areas. The guide values of nutrients in the leaves of the healthy lime and horse chestnut trees was determined.

## Materials and method

Poznań (52°24'30.4"N, 16°56'03.4"E) is the fifth most populated city (over 540,000 inhabitants) in Poland. Its area is the seventh largest (over 261 km^2^) in Poland. Green areas in Poznań form a ring-wedge system, which means that they are arranged into four wedges in natural valleys of the Warta, Bogdanka and Cybina rivers. Apart from forests and green spaces in housing estates, there are more than 270 separate green areas in the city. These are numerous parks, green spaces, allotment gardens, science and research parks, two zoos and 24 cemeteries [35, 36].

**Fig 1:**
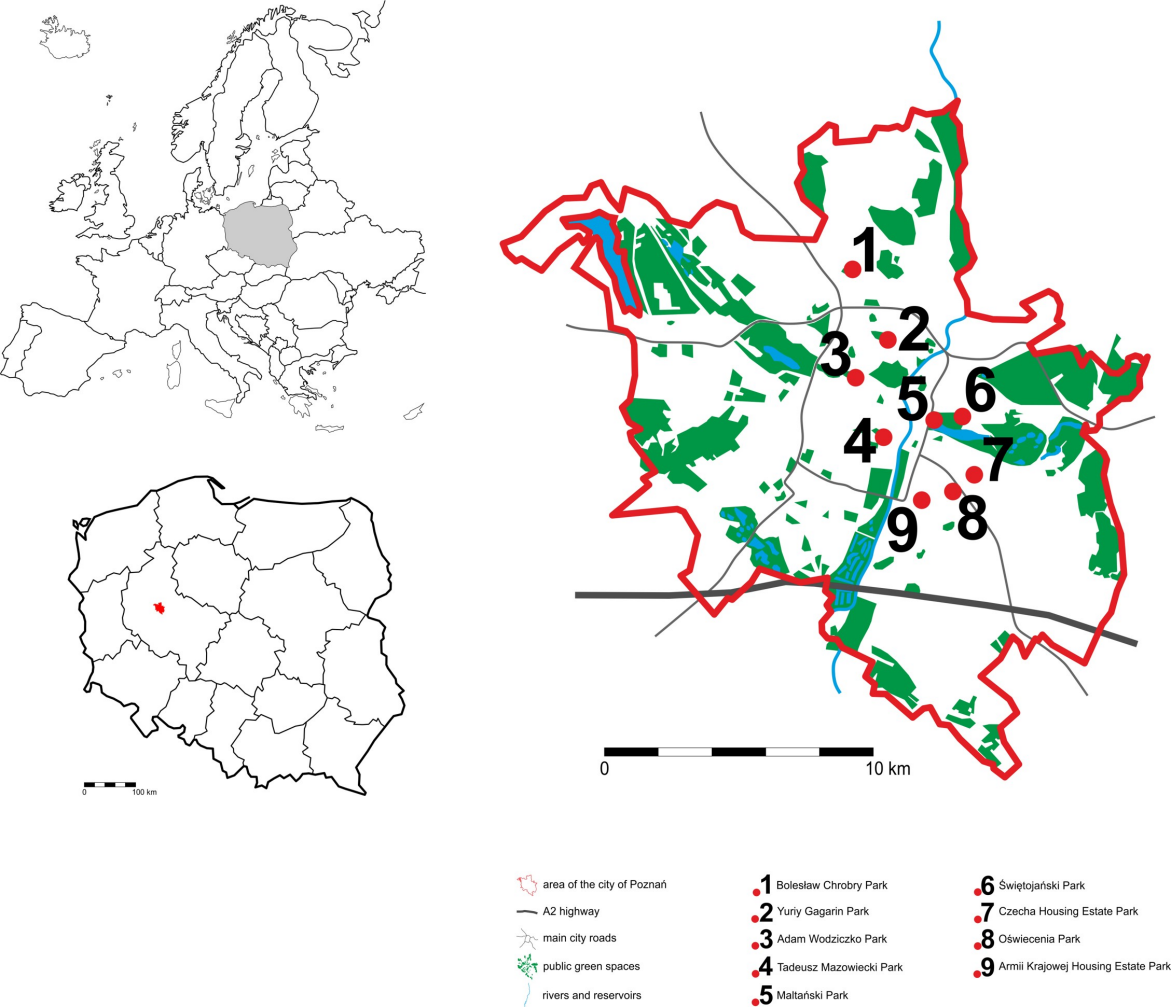
The location of the research sites.

The study was conducted in non-historical public parks in Poznań, used by citizens for recreation and passive rest. *T*. *cordata* Mill. and *A*. *hippocastanum* L. trees can be found in the parks selected for the research (Fig 1).

The research sites were located in different parts of the city–both closer to the centre and industrial plants as well as in housing estates. Table 1 shows detailed locations of the research sites (with division into four sectors: northwest [NW], southwest [SW], northeast [NE] and southeast [SE]), their areas and the number of specimens of each species under study.

**Table 1:** Characteristics of the examined parks in Poznań.

*Sector*	*Number (according to **Fig* 1*)*	*Name of a park*	*Location*	*Area [ha]*	*Number of trees* *(mean age in years)*	
					*T*. *cordata*	*A*. *hippocastanum*
NW	1.	B. Chrobry Park	52°27'20.0"N 16°54'50.2"E	8.7	38 (23.2)	31 (20.1)
	2.	Y. Gagarin Park	52°26'00.9"N 16°56'26.2"E	4.3	46 (29.8)	22 (32.3)
SW	3.	A. Wodziczko Park	52°25'11.4"N 16°54'46.6"E	8.2	110 (61.3)	4 (57.9)
	4.	T. Mazowiecki Park	52°23'57.9"N 16°56'17.3"E	5.1	50 (58.2)	37 (55.3)
NE	5.	Maltański Park	52°24'23.6"N 16°57'20.4"E	15.0	15 (24.1)	14 (24.7)
	6.	Świętojański Park	52°24'26.0"N 16°57'41.7"E	2.3	10 (75.0)	7 (71.7)
SE	7.	Czecha Housing Estate Park	52°23'05.3"N 16°59'07.0"E	5.0	20 (19.8)	6 (19.1)
	8.	Oświecenia Housing Estate Park	52°23'22.9"N 16°57'51.3"E	8.4	101 (21.3)	24 (22.7)
	9.	Armii Krajowej Housing Estate Park	52°22'39.1"N 16°57'18.9"E	8.0	60 (18.6)	48 (20.1)
**SUM:**				**65.0**	**450**	**193**

The health of the trees was assessed with the non-invasive method developed by Kosmala [37], who modified Roloff’s method. It enables assessment of the health of trees exposed to anthropogenic factors in urbanised areas. The health of the whole tree (the crown, trunk and root system) was assessed according to the scale shown in Table 2.

**Table 2:** Points range for each of the criteria.

*Element of tree*	*Criteria*	*Points*
1. Crown condition (*C*_*C*_)	1.1. Crown structure	0–15
	1.2. Crown health condition	0–15
2. Trunk condition (*C*_*T*_)	2.1. Trunk structure	0–15
	2.2. Trunk health condition	0–15
3. Roots condition (*C*_*R*_)	3.1. Base of trunk and structural roots status	0–24
	3.2. Root system status (based on soil observation)	0–16

The health evaluation assessed both the crown structure (the degree of defoliation and the crown health condition) and structure and health status of the trunk (including rubbing bark, sapwood and cracks) as well. The method also includes an assessment of the state of the root system of a tree (butt end state and structural root status based on the observation of the soil). The results are calculated as the health condition of a tree according to the formula: *C = C*_*C*_ *+ C*_*T*_ *+ C*_*R*_. The final stage of the assessment in the above-mentioned method is to determine the phase of vitality, health status and health category of trees according to Table 3.

**Table 3:** Phases of vitality, health condition and health category of the examined trees.

*Points*	*Health condition*	*Vitality phase*	*Health category of a tree [Q]*
100–96	very good	expansion	I
95–76	good		II
75–46	average	reduction	III
45–16	bad	stagnation	IV
15–0	very bad	resignation	V

### Soil analysis

Soil samples from the layer of 0–20 cm were taken using Egner’s stick, while for the deeper layer (20–40 cm), the soil auger was used. The roots of trees reach a depth exceeding 60 cm. Soil samples were taken under healthy, typical and randomly selected trees in the central part of the plantations, in 3 m distance from tree stems. Collected samples were chemically analyzed by the universal method [38]. Extraction of macronutrients (N-NH_4_, N-NO_3_, P, K, Ca, Mg, S-SO_4_), Cl and Na was carried out in 0.03 M CH_3_COOH with a quantitative 1:10 proportion of soil to extraction solution. After extraction, the following determinations were made: N-NH_4_, N-NO3—by microdistillation according to Bremer in Starck’s modification; P–colorimetrically with ammonium vanadomolybdate; K, Ca, Na–photometrically; Mg–by atomic absorption spectrometry (ASA, on Carl Zeiss-Jena apparatus); S-SO4 –nephelometrically with BaCl_2_; Cl–nephelometrically with AgNO_3_. Micronutrients (Fe, Mn, Zn and Cu) and heavy metals (Cd, Pb) were extracted from soil with Lindsay’s solution containing in 1 dm^3^: 5 g EDTA (ethylenediaminetetraacetic acid); 9 cm^3^ of 25% NH_4_ solution, 4 g citric acid; 2 g Ca(CH_3_COO)_2_·2H_2_O. Micronutrients and heavy metals were determined by ASA method. Salinity was identified conductometrically as an electrolytic soil conductivity (EC in mS·cm^-1^), and pH–was determined by potentiometric method (soil : water = 1:2) [39].

### Plant material analysis

Representative samples (fully developed and healthy leaves, on each side of the outer part of the crown) were collected in second half of June 2016. Dust and any impurities were mechanically removed from the leaves surface. Plant material and ground were dried at 45–50°C. In order to assay the total forms of N, P, K, Ca, Mg and Na, the plant material (1 g) was digested in concentrated (96%, analytically pure) sulphuric acid (20 cm^3^) with the addition of hydrogen peroxide (30%, analytically pure). For analyses of total Fe, Mn, Zn, Cu, Cd and Pb the plant material (2,5 g) was digested in a mixture of concentrated nitric (ultra-pure) and perchloric acids (analytically pure) at a 3:1 ratio (30 cm^3^) [40]. After digestion of the plant material, the following determinations were performed: N-total using the distillation method according to Kjeldahl in a Parnas Wagner apparatus; P, colorimetrically with ammonium molybdate, and K, Ca, Mg, Na, Fe, Mn, Zn, Cu, Cd, Pb using flame atomic absorption (on a AAS, on a Carl Zeiss Jena apparatus).Earlier in the laboratory the accuracy of the used methods of chemical analyses and the precision of analytical measurements of heavy metals was tested by means of the analysis of the reference material of branched flour (*Pseudevernia furfuracea*), certified by the IRMM (Institute for Reference Materials and Measurements) in Belgium [41].

### Statistical analysis

Statistical analyses and models were based on discriminant analysis. The analyses showed which environmental variables measured in the leaves and soils at the sites of the two species could affect the health status (Q) of trees in the locations under study. The model was based on canonical variate analysis (CVA), which is the canonical variant of the Fisher linear discriminant analysis (LDA) [42]. The discriminant analysis enabled comparison of the influence of different groups of variables on the health of trees in different locations. The following parameters were included in the analysis: macronutrients (N, N-NH_4_, N-NO_3_, P, K, Ca, Mg, S-SO_4_), micronutrients (Fe, Mn, Zn, Cu, Cl), sodium (Na), heavy metals (Cd, Pb) , pH and soil salinity (EC).

The CVA resulted in four CCA models. A progressive stepwise analysis was applied to find which variables had the biggest influence on the distribution of the health condition of the trees in the locations under study. All variables were assessed. The ones which contributed most to group discrimination based on the p and F values for a particular variable were included in the model. This process was repeated until the p value dropped below 0.05 and the F value dropped below 2 for the variable under analysis. The significance limit was determined by means of the Monte Carlo permutation test (999 permutations). The Canoco for Windows package (Canoco for Windows 4,5, CanoDraw for Windows and WCanoIMP) and the Microsoft Excel spreadsheet were used for statistical analyses and graphic elements.

## Results

Apart from horse chestnut tree site No. 7, the soils at most of the sites were slightly alkalised. Their pH ranged from 7.16 to 7.93 (horse chestnut trees) and from 7.13 to 8.04 (lime-trees) (Table 4). It is most likely that the excessive pH was caused by the high content of calcium as the alkaline cation. It ranged from 794.1 to 5,513.6 mg·dm^-3^ (horse chestnut trees) and from 1,009 to 6,092.3 mg·dm^-3^ (lime-trees). The soils at the sites were not excessively salinated. The soil salinity did not exceed 0.28 mS·cm^-1^ (horse chestnut trees) and 0.38 mS·cm^-1^ (lime-trees). In general, the content of ballast ions: sodium and chlorides were within the permissible limits. The content of the following macro- and micronutrients in the soil (0–40 cm) was respectively characterised by the lowest variability: magnesium (CV = 44.9%) and iron (CV = 43.6%) at the sites of the horse chestnut trees, and calcium (38.7%) and copper (38.9%) at the sites of the lime-trees. The salinity variability coefficients amounted to 40.8% (lime-trees) and 51.7% (horse chestnut trees). There were mostly standard amounts of micronutrients (iron, manganese, zinc, copper and chlorides) in the soils at the sites of lime-trees. The soils were not excessively contaminated with cadmium or lead. There were trace amounts of nitrogen content, normal content of potassium and magnesium and low content of phosphorus and sulphate sulphur (S-SO_4_).

**Table 4:** Chemical properties of soils and coefficients of their variability on sites of *T*. *cordata* Mill. and *A*. *hippocastanum* L.

*Location*	*Depth (cm)*	*N-* *NH*_*4*_	*N-* *NO*_*3*_	*P*	*K*	*Ca*	*Mg*	*S-SO*_*4*_	*Fe*	*Mn*	*Zn*	*Cu*	*Cl*	*Na*	*Cd*	*Pb*	*pH*	*EC* mS∙cm^-1^
									*mg·dm*^*-3*^									
***Tilia cordata* Mill.**																		
1.	0–20	tr.	tr.	40.1	60.1	1498.0	118.4	0	117.7	28.4	13.0	3.58	8.87	9.20	0.46	5.86	7.34	0.09
	21–40	tr.	tr.	83.5	36.9	5258.5	100.3	5.0	28.4	10.8	10.9	2.87	9.20	10.90	0.45	3.00	7.82	0.14
2.	0–20	tr.	tr.	74.1	43.7	2343.9	145.8	6.3	129.3	60.7	4.7	2.58	11.38	13.80	0.42	7.85	7.84	0.11
	21–40	tr.	tr.	9.8	136.8	3710.0	155.4	5.6	30.1	15.4	5.5	1.74	22.40	21.90	0.41	4.17	7.98	0.13
3.	0–20	tr.	tr.	13.7	90.3	5615.2	149.3	4.2	46.6	7.9	9.2	2.63	13.71	12.90	0.45	4.68	7.69	0.18
	21–40	tr.	tr.	7.4	83.0	5392.0	207.7	29.2	25.1	10.2	13.8	2.66	10.51	15.60	0.46	7.87	7.84	0.18
4.	0–20	tr.	tr.	7.9	135.0	4136.6	190.9	53.0	49.9	7.8	22.2	5.08	9.53	21.00	0.42	10.17	7.84	0.18
	21–40	tr.	tr.	8.4	75.9	3070.7	132.9	29.0	97.4	8.5	37.6	5.29	7.25	20.60	0.53	14.39	7.57	0.18
5.	0–20	tr.	tr.	20.0	233.6	5833.6	309.9	21.3	31.5	17.2	4.8	2.57	24.29	54.90	0.45	3.46	7.59	0.38
	21–40	tr.	tr.	45.6	166.6	5757.4	161.8	18.8	42.6	5.0	26.1	5.55	14.83	18.80	0.53	14.95	7.80	0.20
6.	0–20	tr.	tr.	6.5	152.9	2898.1	374.8	4.7	89.7	14.2	14.9	3.93	10.29	15.30	0.48	6.18	7.33	0.13
	21–40	tr.	tr.	12.2	216.7	3081.6	282.7	1.9	88.3	17.2	18.6	5.41	9.64	10.70	0.46	7.69	7.16	0.15
7.	0–20	tr.	tr.	54.1	64.5	1000.9	120.8	3.3	143.1	52.2	11.8	3.04	8.55	7.30	0.45	8.52	7.13	0.09
	21–40	tr.	tr.	64.3	121.8	2860.2	238.6	10.9	93.7	16.7	14.2	3.36	36.84	14.30	0.42	6.43	7.41	0.11
8.	0–20	tr.	tr.	29.2	118.6	5773.6	159.6	12.0	33.2	10.7	5.6	2.24	8.33	11.00	0.47	10.93	7.67	0.16
	21–40	tr.	tr.	15.1	35.5	5443.3	167.7	32.9	38.8	7.0	5.7	1.86	7.90	10.40	0.42	3.63	7.84	0.16
9.	0–20	tr.	tr.	8.9	62.2	6043.3	136.5	9.2	30.8	9.2	8.9	2.17	6.82	8.40	0.48	3.31	7.65	0.10
	21–40	tr.	tr.	23.3	186.9	6092.3	142.5	31.6	28.2	9.0	5.2	1.73	10.62	11.90	0.472	2.848	8.04	0.14
CV in %	0–20	0	0	78.7	53.5	48.6	45.1	121.6	57.7	81.7	50.9	29.0	43.8	81.5	4.6	38.6		54.2
	21–40	0	0	87.9	51.9	27.3	30.3	65.3	55.7	37.1	66.8	45.1	63.2	28.1	9.1	60.5		17.9
	Mean	0	0	83.8	52.9	38.7	39.1	90.8	60.4	87.3	65.7	38.9	58.2	64.5	7.3	51.5		40.8
	0–20	0	0	22.3	15.4	1898.5	85.5	15.4	43	18.9	5.4	0.9	4.69	13.9	0.02	2.62	0.23	0.09
SD	21–40	0	0	26.3	12	1235.7	53.4	12.0	29.2	4.1	10.2	1.5	9.07	4.2	0.04	4.37	0.27	0.03
	Mean	0	0	24.4	14.1	1630.9	71.6	14.1	38.4	15	8.5	1.3	7.46	10.3	0.03	3.61	0.26	0.06
***Aesculus hippocastanum* L.**																		
1	0–20	tr.	tr.	17.1	117.5	4194.3	220.7	0.27	94.31	34.86	9.09	2.57	7.68	10.70	0.13	4.59	7.31	0.07
	21–40	tr.	tr.	12.2	36.1	3792.6	171.1	10.46	84.49	17.39	18.66	5.03	7.04	11.10	0.34	7.83	7.72	0.09
2	0–20	tr.	tr.	87.7	89.6	1164.2	117.5	0.13	126.11	60.92	12.82	4.14	11.82	14.10	0.31	12.46	7.50	0.08
	21–40	tr.	tr.	8.9	100.6	3827.3	120.1	1.33	39.56	6.87	5.13	2.10	11.84	17.70	0.21	6.01	7.78	0.17
3	0–20	tr.	tr.	12.7	128.8	5005.6	133.9	29.95	41.16	6.39	13.33	3.65	8.65	13.10	0.28	7.45	7.74	0.18
	21–40	tr.	tr.	29.7	260.1	5267.3	302.5	151.10	64.32	21.74	16.97	7.11	29.22	113.40	0.36	14.96	7.76	0.28
4	0–20	tr.	tr.	11.8	26.4	4981.6	110.1	47.50	59.14	4.52	41.78	5.83	8.76	15.10	0.42	28.38	7.72	0.18
	21–40	tr.	tr.	14.1	48.2	4424.1	161.2	38.31	52.34	8.15	27.94	7.54	6.18	13.30	0.31	12.53	7.83	0.17
5	0–20	tr.	tr.	6.5	45.2	4559.6	137.5	18.97	80.01	9.15	4.88	2.56	10.07	16.10	0.26	3.26	7.86	0.13
	21–40	tr.	tr.	5.1	19.7	2840.6	166.9	0.0	101.16	16.57	4.01	2.23	6.93	13.60	0.25	3.31	7.93	0.07
6	0–20	tr.	tr.	76.7	166.5	1586.6	237.3	0.0	123.67	16.84	30.52	6.62	10.29	9.20	0.35	14.04	7.38	0.09
	21–40	tr.	tr.	139.1	127.7	862.6	123.2	4.42	156.92	52.18	27.87	10.31	13.26	9.10	0.31	33.46	7.31	0.06
7	0–20	tr.	tr.	13.7	51.3	2472.3	118.3	10.60	85.81	5.84	6.78	2.65	6.93	12.70	0.36	6.76	6.52	0.19
	21–40	tr.	tr.	8.4	63.3	2190.1	380.1	0.0	119.38	31.01	27.83	1.17	16.63	18.60	0.32	4.29	5.88	0.03
8	0–20	tr.	tr.	16.1	80.3	5513.6	186.1	9.49	48.62	7.36	12.57	6.12	8.33	9.10	0.44	6.67	7.28	0.18
	21–40	tr.	tr.	6.5	27.7	5068.5	253.5	15.67	61.86	7.43	5.15	2.34	9.64	18.30	0.46	5.59	7.57	0.13
9	0–20	tr.	tr.	40.1	47.1	794.1	101.3	0.0	120.25	45.64	18.45	3.56	13.26	11.50	0.44	10.73	7.16	0.10
	21–40	tr.	tr.	83.5	118.2	2266.6	164.6	4.98	53.13	25.85	27.55	3.55	8.55	12.60	0.62	11.17	7.75	0.12
CV in %	0–20	0	0	91.5	52.3	51.9	31.4	120.3	35.4	92.0	68.1	36.2	20.1	18.9	28.6	68.3		35.7
	21–40	0	0	128.2	79.8	40.7	40.7	182.8	44.1	65.6	56.2	63.9	56.3	123.7	32.5	79.5		58.7
	Mean	0	0	111.3	71.8	50.5	44.9	166.5	43.6	82.7	65.7	57.3	52.1	122.6	34.3	77.3		51.7
SD	0–20			28.7	43.76	1746.39	47.55	15.62	30.61	19.58	11.37	1.52	1.92	2.34	0.10	7.16	0.38	0.05
	21–40			43.8	71.06	1380.64	83.38	45.96	35.92	13.65	10.07	2.94	6.84	31.3	0.11	8.76	0.6	0.07
	Mean			37.06	59.07	1574.25	72.93	34.86	33.47	16.88	10.75	2.35	15.19	23.12	0.11	8.01	0.5	0.06

The leaves of horse chestnut trees contained the following amounts of macronutrients (% d. m.): N 2.38%-4.71%, P 0.24%-0.46%, K 1.13%-2.31%, Ca 1.05%-2.12%, Mg 0.16%-0.42% and S 0.12%-0.23% (Table 5). The following content of micronutrients were determined (in d.m.): Fe 89.8–198.8 mg∙kg^-1^, Mn 10.2–95.0 mg∙kg^-1^, Zn 17.6–33.1 mg∙kg^-1^ and Cu 7.36–19.61 mg∙kg^-1^. The following content of macronutrients were measured in the leaves of lime-trees (% d. m.): N 2.45%-3.64%, P 0.23%-0.42%, K 1.52%-2.86%, Ca 1.43%-2.04%, Mg 0.19%-0.49%, S 0.17%-0.32%. The content of micronutrients looked as follows: Fe 92.7–174.3 mg∙kg^-1^ d. m., Mn 12.4–113.5 mg∙kg^-1^ d. m., Zn 19.9–26.9 mg∙kg^-1^ d. m. and Cu 7.31–15.38 mg∙kg^-1^ d. m. The coefficients of variation (CV) referring to the content of components in the leaves were generally lower than the values referring to the content of components measured in the soil. The content of manganese and sodium in the leaves exhibited significantly greater variability, i.e. CV 80.9% and 49.5%, respectively in the lime-trees and 78% and 106.1% in the horse chestnut trees. There was generally low variability in the content of cadmium and lead in both species (except Pb in the case of horse chestnut).

**Table 5:** Chemical composition of leaves and coefficients of their variability.

*Location*	*N*	*P*	*K*	*Ca*	*Mg*	*S*	*Fe*	*Mn*	*Zn*	*Cu*	*Na*	*Cd*	*Pb*
***Tilia cordata* Mill.**													
1.	2.66	0.38	1.99	1.73	0.45	0.28	140.4	49.2	25.8	15.38	0.01	0.89	5.26
2.	2.80	0.27	1.52	1.52	0.19	0.24	137.6	60.7	22.4	9.28	0.02	0.90	4.31
3.	2.45	0.42	2.86	2.02	0.35	0.25	154.4	12.4	23.8	8.36	0.01	1.14	6.49
4.	3.15	0.28	1.57	1.69	0.49	0.17	161.2	19.7	22.1	11.92	0.01	0.93	5.13
5.	2.85	0.24	1.65	1.85	0.21	0.21	141.7	21.0	25.0	9.61	0.01	1.17	5.65
6.	3.22	0.30	1.60	1.43	0.35	0.19	174.3	33.5	20.2	9.79	0.02	1.04	5.66
7.	3.50	0.27	1.88	1.48	0.19	0.26	100.2	113.5	26.9	7.31	0.02	1.14	4.38
8.	3.64	0.23	1.70	1.83	0.21	0.27	92.7	16.1	22.7	10.92	0.01	1.16	5.07
9.	3.29	0.26	1.61	2.04	0.30	0.32	133.0	17.0	19.9	8.56	0.01	1.31	6.45
Mean	3.06	0.30	1.82	1.73	0.30	0.24	137.3	38.1	23.2	10.13	0.01	1.08	5.38
CV (%)	12.2	20.3	21.7	12.1	34.5	17.9	18.2	80.9	9.7	22.3	49.5	12.7	13.6
***Aesculus hippocastanum* L.**													
1.	2.87	0.29	1.49	1.89	0.23	0.16	105.6	29.5	24.2	13.35	0.01	1.38	5.92
2.	3.85	0.27	1.26	2.08	0.34	0.13	198.8	83.8	33.1	18.21	0.05	1.65	8.46
3.	4.71	0.24	1.61	1.38	0.16	0.12	134.9	10.2	18.4	7.36	0.01	1.31	5.64
4.	2.94	0.32	1.37	1.71	0.42	0.23	181.2	24.9	27.4	16.02	0.01	1.46	7.61
5.	3.15	0.24	1.44	2.12	0.28	0.15	157.2	12.3	25.2	19.61	0.01	1.61	8.43
6.	2.38	0.46	2.31	1.05	0.21	0.16	174.8	35.5	17.6	11.74	0.01	1.33	7.74
7.	3.36	0.29	1.56	1.61	0.31	0.14	151.5	95.0	19.7	11.25	0.01	1.51	6.87
8.	2.45	0.29	1.13	1.85	0.27	0.14	89.8	16.4	25.0	12.58	0.01	1.69	6.99
9.	2.52	0.26	1.42	2.11	0.24	0.15	133.8	27.7	24.0	16.18	0.01	1.82	8.01
Mean	3.14	0.30	1.51	1.76	0.27	0.15	147.5	37.2	23.8	14.03	0.01	1.53	7.30
CV (%)	22.7	21.0	20.9	19.5	26.5	19.4	22.7	78.0	19.2	25.7	106.1	10.8	13.2

Macroelements and Na expressed in % d.m., microelements and heavy metals: in mg∙kg^-1^ d.m.

**Fig 2:**
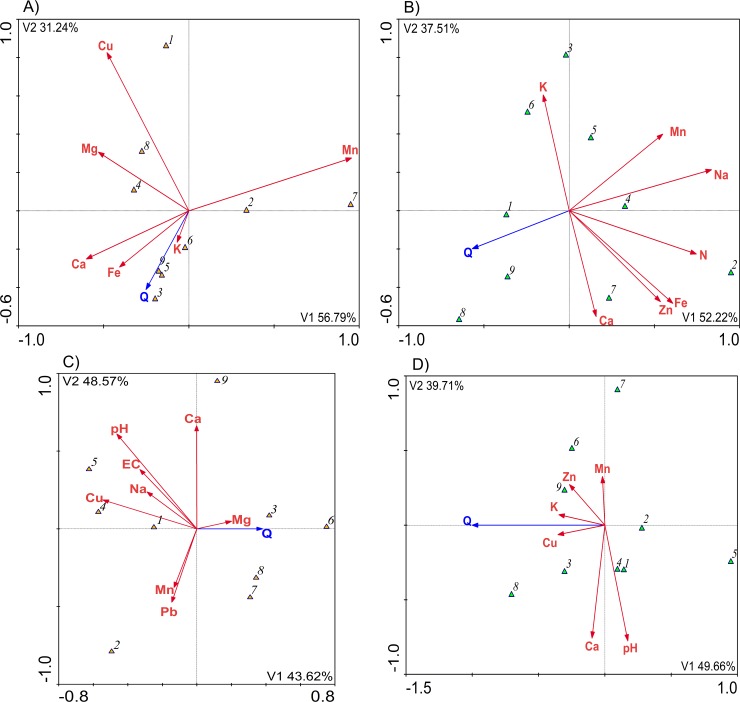
A CCA model. The dependencies between the parameters in the leaves of: A) lime-trees, B) horse chestnut trees; and in the soil at the sites of: C) lime-trees, D) horse chestnut trees and the health of the trees (Q) at their locations (items numbered as in Table 1).

The statistical analysis showed a positive correlation between the content of Ca and Mn in the leaves of lime-trees and at all soil levels at their sites. This might indicate a significant dependency between them (Table 6). As far as the leaves and the soil profile (0–40 cm) at the sites of horse chestnut trees are concerned, there were dependencies between the content of K and Cd (Table 7). Apart from that, the CCA (Fig 2) showed a positive correlation between the content of Ca in the lime-tree leaves and the health of the trees. As far as the horse chestnut trees are concerned, there was a positive dependency between the content of potassium in the soil and the health of the trees.

**Table 6:** Pearson correlation coefficients between the content of macro- and microelements, Na and heavy metals in the soil and in lime-tree leaves.

*Depth (cm)*	*N*^a^	*P*	*K*	*Ca*	*Mg*	*S-SO*_*4*_^b^	*Fe*	*Mn*	*Zn*	*Cu*	*Cl*	*Na*	*Cd*	*Pb*
0–20	0 ^n.s.^	-0.10 ^n.s.^	-0.20 ^n.s.^	0.81 **	0.01 ^n.s..^	-0.63 *	-0.11 ^n.s.^	0.86 ****	-0.11 ^n.s.^	0.45 ^n.s.^	-0.15 ^n.s.^	0.65 *	-0.70 ***	-0.11 ^n.s.^
21–40	0 ^n.s.^	0.15 ^n.s.^	-0.37 ^n.s.^	0.87 **	-0.31 ^n.s.^	0.19 ^n.s.^	0.15 ^n.s.^	0.70 ***	0.07 ^n.s.^	0.10 ^n.s.^	0.16 ^n.s.^	0.12 ^n.s.^	0.07 ^n.s.^	0.15 ^n.s.^
0–40	0 ^n.s.^	0.04 ^n.s.^	-0.36 ^n.s.^	0.91 ****	-0.13 ^n.s.^	-0.32 ^n.s.^	-0.01 ^n.s.^	0.88 ****	0.01 ^n.s..^	0.25 ^n.s.^	-0.09 ^n.s.^	0.41 ^n.s.^	-0.30 ^n.s.^	-0.01 ^n.s.^

Correlation relevant at

* α 0,1

** α 0,01

*** α 0,05

**** α 0,001; n.s.–not significant

^a^ sum of N-NH_4_ i N-NO_3_

^b^ content of S in leaves

**Table 7:** Pearson correlation coefficients between the content of macro- and microelements, Na and heavy metals in the soil and in horse chestnut tree leaves.

*Depth* *(cm)*	*N*^a^	*P*	*K*	*Ca*	*Mg*	*S-SO*_*4*_ ^b^	*Fe*	*Mn*	*Zn*	*Cu*	*Cl*	*Na*	*Cd*	*Pb*
0–20	0.09 ^n.s.^	0.46 ^n.s.^	0.66 *	-0.03 ^n.s.^	-0.46 ^n.s.^	0.56 *	0.44 ^n.s.^	0.36 ^n.s.^	0.01 ^n.s.^	-0.14 ^n.s.^	-	0.29 ^n.s.^	0.51 ^n.s.^	0.28 ^n.s.^
21–40	0.27 ^n.s.^	0.72 **	0.38 ^n.s.^	0.23 ^n.s.^	-0.17 ^n.s.^	-0.17 ^n.s.^	0.08 ^n.s.^	0.11 ^n.s.^	-0.52 ^n.s.^	-0.39 ^n.s.^	-	-0.16 ^n.s.^	0.48 ^n.s.^	-0.02 ^n.s.^
0–40	0.20 ^n.s.^	0.70 ^n.s.^	0.56 *	0.09 ^n.s.^	-0.42 ^n.s.^	0.02 ^n.s.^	0.31 ^n.s.^	0.38 ^n.s.^	-0.27 ^n.s.^	-0.34 ^n.s.^	-	-0.14 ^n.s.^	0.57 *	0.14 ^n.s.^

Correlation relevant at

* α 0,01

** α 0,05; n.s.–not significant

^a^ sum of N-NH_4_ + N-NO_3_

^b^ content of S in leaves

The CCA models showed dependencies between the parameters in the leaves and soil at the research sites. The parameters determined in the leaves were significantly correlated with the health of the trees (Table 8, Fig 2A and 2B). The content of K, Ca and Fe in the lime leaves was positively correlated with the health of the trees (Q). The increase in the content of these nutrients in the leaves may have significantly affected the condition of the trees (Fig 2A). The opposite tendency was observed in the horse chestnut leaves (Fig 2B), as most of the parameters may have negatively affected the health of the trees. Apart from that, there was a very distinct antagonism between the content of Mn and the content of Ca and Fe in the lime- trees.

**Table 8:** Adjustment coefficients for the models presented in (Fig 2A–2D).

*Variables*	*p-value*	*F-value*	*% Expl*.	*Variables*	*p-value*	*F-value*	*% Expl*.
**A**				**B**			
Mn	0.001	3.996	15.72	Q	0.001	6.885	15.70
Cu	0.001	4.593	12.69	Mn	0.001	4.590	12.61
Q	0.001	6.772	12.61	Fe	0.001	3.567	12.49
Mg	0.002	12.698	10.85	K	0.002	3.994	11.27
Ca	0.001	28.394	9.94	N	0.002	5.470	10.58
Fe	0.001	3.357	9.65	Zn	0.004	9.485	10.52
K	0.001	5.419	8.44	Ca	0.002	15.900	9.94
				Na	0.012	5.018	6.24
**C**				**D**			
Ca	0.001	6.541	10.42	Q	0.001	6.458	14.99
pH	0.001	3.541	10.11	pH	0.002	4.942	12.62
Q	0.002	8.457	10.07	Ca	0.001	12.594	11.73
Cu	0.004	3.233	8.45	Mn	0.003	5.364	8.45
EC	0.002	4.842	7.45	Cu	0.005	3.771	8.11
Pb	0.002	3.235	7.23	Zn	0.007	7.512	8.01
Mn	0.003	3.133	6.27	K	0.009	3.179	7.69
Mg	0.007	9.846	6.21				
Na	0.045	2.177	5.47				

The negative effect of Mn contained in the leaves on the health of trees (Q) was a certain regularity in the models under study. It is particularly interesting due to the slightly alkaline pH of the soils. This indicates the need to conduct further research on the toxicity of this component to plants growing in cities.

The research showed that the nutrition of trees depended on their location. For example, the highest content of potassium in both tree species was found in Wodziczki and Świętojański Parks. The highest content of manganese was measured in the leaves collected from the trees growing in Gagarina and Czecha Parks.

The analysis of the mathematical models (Fig 2) for the chemical composition of the soil at the sites of lime and horse chestnut trees showed that the trees growing on soils with higher Mn content (Gagarin Park) were categorised into a lower health class Q. The healthiest trees were found in Wodziczki and Świętojański Parks. Their health (Q) was positively correlated with the Mg content in the soil samples collected from the lime-tree sites and with the K and Cu content in the soils collected from the horse chestnut tree sites (Fig 2C and 2D). The trees in Maltański Park were the least healthy (Q). Their condition may have been influenced by the chemical properties of the soil. The soil salinity and Na content may have negatively affected the health of lime-trees.

## Discussion

### Soil analysis

Soils in urban areas are heterogeneous formations, which were strongly transformed by humans. They have defective properties for the growth and development of plants. Anthropogenic transformations of these soils include changes in their physical properties: the deterioration of porosity and structure, compaction, mixing of the natural system of levels and contamination with various pollutants. These soils are also characterised by various negative changes in their chemical composition, which significantly influence the growth, development and appearance of plants [43–45]. The conditions of plants’ growth are usually not optimal [46,47]. Therefore, the urban environment significantly affects the condition of green spaces in cities.

The research showed that the alkalisation of soils with excessive contents of alkaline cations, including calcium, was an important problem in urban habitats. Other authors also reported the problem of excessive calcium content in the soils in Poznań [48–50]. The accumulation of this component in urban soils may be caused by the deposition of dust produced as a result of burning solid fuels and by leaving construction debris in the soil (this mainly applies to green areas in housing estates) [51]. The excess of calcium in soil may affect plants’ health more negatively than soil salinity [52]. Alkalisation deteriorates the availability of phosphorus (chemical sorption with magnesium and calcium ions) and micronutrients (Fe, Mn, Zn, Cu and Ni–in consequence of their lower solubility and antagonism against Ca). This may negatively affect the growth of plants and increase their susceptibility to diseases or even cause their death [48]. The problem of alkalisation of soils in cities was confirmed by Bosiacki et al. [53], who found that the pH of 46.7% of the samples collected from soils adjacent to roads in Poznań ranged from of 7.4 to 8.5. Researchers (e.g. Kleiber [50]) indicated the problem of alkaline pH in the soils under old lime-trees in Luboń (Poznań suburbs), where it ranged from 7.30 to 10.95, depending on the location. Other scientists confirmed the problem of alkalisation of urban soils in their study on Nadolnik Park in Poznań, where as much as 69% of the soil samples were alkaline [49]. There were similar results of studies conducted in other countries (including Greece, China), where researchers also found alkaline soil pH in urban areas [54,55]. The research conducted on green spaces in Warsaw (the capital of Poland) and Wrocław showed that the pH of soils decreased with the distance from roads [56,57]. This fact might point to the influence of NaCl, which is used to deice roads. This compound has strong alkalising effect on soil due to the formation of sodium hydroxide.

There were standard amounts of magnesium in the soil at most of the sites with lime and horse chestnut trees, i.e. on average 183.09 mg dm^-3^ and 178.11 mg dm^-3^, respectively. The content of this component in urban soils is strongly diversified. The following contents were measured in other studies: Singapore 313–631 mg kg^-1^, Baltimore (USA) 21–388 mg kg^-1^, Chicago (USA) 641–797 mg kg^-1^, Kielce (Poland) 1,000–4,900 mg kg^-1^, Riga (Latvia) 1,472–2,960 mg kg^-1^ [24,58–60]. Excessive calcium content in soils is an unfavourable phenomenon. In our study the average content amounted to 4,211.54 mg dm^-3^ Ca (lime-trees) and 3,378.42 mg dm^-3^ Ca (horse chestnut-trees). It had very negative effect due to the extension of the quantitative ratio between Ca and Mg, which should optimally be 6–8:1. For example, at horse chestnut tree site IV the ratio was as high as 45.2:1 (0–20 cm). By contrast, the study conducted in Singapore showed that the average Ca: Mg ratio was 2.9:1, and yet it did not negatively affect plants’ uptake of calcium, their development and health.

In general, the soils under study were characterised by very low salinity (EC–electrical conductivity), i.e. 0.03–0.38 mS·cm^-1^. These results positively correspond with the findings of earlier studies [50]. The results of our study are also reflected in the results of chemical analyses conducted in Nadolnik Park in Poznań, where salinity was also low (0.08–0.74 mS·cm^-1^) [49]. There was a similar salinity level in Maribor (Slovenia) [61]. Salinity is more strongly affected by anions because they exhibit lower physicochemical sorption in the sorption complex (it is usually negatively charged in soils in the temperate climate). Increased soil salinity in urban areas is caused by the use of road salt containing NaCl [51]. However, the content of sodium measured in the profiles analysed in our study was low. The excess of this ballast ion in soils is negative, because it deteriorates their structure and causes strong alkalisation. Apart from that, it may disorder plants’ uptake of other cations, e.g. potassium, and thus it may affect the condition of tree stands [48].

Quantitative relations between the components were disordered in the soils. These observations positively correspond to data in reference publications. There were trace content of nitrogen at most of the sites in our study. So far the research conducted in areas exposed to anthropogenic pressure in Poznań showed that the content of N-NH_4_ did not exceed 11 mg dm^-3^, and the maximum content of N-NO_3_ was 36 mg·dm^-3^ [48]. Other scientists also found trace amounts of nitrogen in the soil in Nadolnik Park in Poznań [49]. According to reference publications, there are different, species-dependent contents of phosphorus in soil, e.g. 12.5 mg kg^-1^ for maize, 14.3 mg kg^-1^ for soy, 19 mg kg^-1^ for wheat and as high as 620 mg kg^-1^ for beech-trees [62,63]. Most of the locations were characterised by phosphorus deficit. However, according to other authors, the content of this component in soils in Poznań is strongly diversified. For example, Golcz et al. [49] found that the component was deficient in most of the researched sites in Nadolnik Park. According to Breś [48], the content of phosphorus depended on the research site (9–163 mg dm^-3^). Apart from that, the researcher found local potassium deficiency in soils and strong phosphorus reversion. These observations were also confirmed in different study [50].

The average potassium content measured in the soils in our study (average density of mineral soils 1.5 kg dm^-3^) amounted to 112.28 mg dm^-3^ (lime-trees) and 86.35 mg dm^-3^ (horse chestnut trees). The potassium content measured in urban soils in Singapore was 27–130 mg kg^-1^ [59]. There were similar amounts of potassium measured in urban soils in Baltimore, (USA) (12–280 mg kg^-1^) and Chicago (USA) (140–199 mg kg^-1^) [60,64]. When the potassium content drops below 200 mg kg^-1^, it may cause stress reactions in trees and deteriorate their health [24]. However, our study did not confirm this observation. Although the content of potassium was much lower, the lime and horse chestnut trees were in very good or good health and they were expanding. Our research clearly showed that the health of the species under study was positively correlated with the potassium content. As the content of this component increased in the soil, the health of the horse chestnut trees improved. At the same time, the increase in the potassium content in the lime leaves was positively correlated with the health of these trees.

Sulphur deficit in soil may negatively affect the growth of plants due to the reduction of their photosynthetic activity [31]. The research showed high variation in the content of this component in the soil (0–151.10 mg S-SO_4_ dm^-3^) and very high coefficients of variation (CV), i.e. 90.8% (lime-trees) and 166.5% (horse chestnut trees). Earlier studies conducted in Poznań showed that the content of sulphur in the soil did not exceed 19 mg dm^-3^ [48]. There were larger amounts of this component at individual sites. There were similar amounts of sulphur in Beijing (China), where they ranged from 14.7 to 189.9 mg dm^-3^ at different sites [65]. The content of sulphur in the soil decreased as the depth increased. However, the researchers observed multidirectional tendencies of variation in their study [65].

Our study revealed noticeable differences in the content of micronutrients in the soil. Kozik et al. [66] conducted research in Nadolnik Park in Poznań and found low contents of micronutrients and heavy metals. Kleiber [50] measured standard contents of metal micronutrients (Fe, Zn, Cu and Mn) in urban soils in Luboń (Poland). In general, the soils at the sites were abundant in zinc (4.01–68.1 mg dm^-3^). There were much larger amounts of this component measured in the soils in Sydney (Australia), i.e. 207.39–10,253.99 mg kg^-1^ [67], and in Paris (France), i.e. 106.95–174.84 mg kg^-1^ [68]. We can say that the soils in almost all big Polish cities (including Wrocław, Gdańsk, Łódź and Bydgoszcz) are contaminated with zinc [68–70]. The maximum permissible limit is 200 mg kg^-1^ [71]. The probable causes of such high soil contamination are oil spills and the wear of car tyres [67,70].

In general, there were standard content of copper found in the soils in our study. According to Kabata-Pendias [31], copper deficit may cause adverse changes in water metabolism and deteriorate the flowering of plants. Excessive copper content largely limits the uptake of Zn and Fe by plants, thus causing chlorosis. Earlier studies showed considerably diversified copper content in Poznań soils (1–1,800 mg kg^-1^), where the concentration tended to increase towards the city centre [70]. The accumulation of Cu in the upper soil layers may be particularly significant. The content of this component decreases as the depth of the profile increases [72]. High contamination of the soil environment with copper is strongly correlated with car traffic intensity [68]. According to Lis and Pasieczna [70], the content of copper in contaminated soils exceeds 20 mg Cu kg^-1^. The content of copper in soils in Paris (France) ranged from 44.50 to 105.23 mg kg^-1^ [68].

In our study the content of iron in the soil at the sites of lime and horse chestnut trees amounted to 25.1–156.92 mg dm^-3^. Łukasiewicz [72] found average and optimal content of iron, i.e. about 5.4 mg 100 g^-1^. The average iron content measured in the soils in Upper Silesia (Poland) was 4,484.5 mg kg^-1^. The component was mostly accumulated in the topsoil layers with high content of organic substances [73].

In our study the content of manganese in the soil was considerably diversified. It ranged from 5.0 to 60.7 mg dm^-3^ at the sites of the lime-trees and from 5.84 to 60.92 mg dm^-3^ at the sites of the horse chestnut trees. The manganese content up to 25 mg dm^-3^ could be regarded as standard. Manganese deficit in the soil may result in excessive growth of lateral roots, inhibit elongation growth and reduce frost resistance [31]. Earlier studies conducted in Poznań at the sites of horse chestnut trees showed normal or reduced Mn amounts, on average 2 mg 100 g^-1^ (in the 0–30 cm layer) [72]. Elevated manganese content in the soil may mostly result from the burning of waste and coal and from the metallurgical industry. There was high site-dependent variation in the manganese content both in our study and in the research conducted by Łukasiewicz [72].

The accumulation of heavy metals in soil and plants indicates pollution of the atmosphere with dust and gases [52]. In general, there are much larger contents of heavy metals in areas bordering busy roads than at other sites. This thesis was confirmed by Wong et al. [74], who said that excessive traffic (car fumes) was the main source of lead in urban green spaces. The analysis of the content of heavy metals in soils in Warsaw showed their highest accumulation in the topsoil. The content was lower in the samples collected from greater soil depths [69]. However, Horváth et al. [44] observed that the concentration of heavy metals in soil tended to increase with depth both in urban and suburban areas.

The content of cadmium was low at all the sites, i.e. 0.13–0.62 mg dm^-3^. The content of lead was usually low. Only at sites IV and VI the content of this metal was elevated, i.e. 28.38 and 33.46 mg dm^-3^, respectively. Kleiber [50] found low contents of heavy metals in soils in Luboń. The study conducted in Nadolnik Park in Poznań in 2014 also showed that the average content of cadmium was low (0.55–0.75 mg dm^-3^), whereas the content of lead was higher (10.3–14.9 mg dm^-3^) [66]. This relatively high variation in the lead content in the soil may indicate that the metal content depended on the traffic intensity at a specific site. The studies conducted in Wrocław and Paris led to similar conclusions–the soils located near busy roads were more polluted with heavy metals [56,68]. Bosiacki et al. [53] conducted research in Poznań and found different coefficients of variation for lead (CV = 110.78%) and cadmium (CV = 17.5%). In our study the content of cadmium at the sites of the lime-trees amounted to 0.41–0.53 mg∙dm^-3^, whereas the content of lead ranged from 2.85 to 14.95 mg∙dm^-3^. The contents of these heavy metals and their coefficients of variation (7.3% and 51.5% respectively) can be regarded as low. The results of our study are in line with other authors’ findings [24,59,60].

### Leaves analysis

The content of nitrogen in plants largely depends on the species [75,76]. According to Kabata-Pendias [31], the average nitrogen content in plants is 0.5–4%. This nutrient significantly affects the growth of plants. In our study the average content of nitrogen in horse chestnut leaves was 3.14% d.m. The content of nitrogen in horse chestnut leaves in Nadolnik Park was lower, i.e. 2.66% [49].

The average content of phosphorus in leaves is 0.1–2.5% [31]. The content of phosphorus in the leaves of the trees at the research sites was normal, i.e. 0.24–0.46% d.m. (horse chestnut trees) and 0.23–0.42% (lime-trees). Appropriate uptake of this component is important for plants’ energy. It has positive influence on the development of the root system, and it increases resistance to frost in winter and drought in summer. The content of phosphorus in the leaves of the trees in Poznań ranged from 0.18% to 0.32% [49], whereas in Wrocław the average content was 0.96% [77].

The average potassium content in plants ranges from 0.3% to 10% [78]. In our study the content of this nutrient ranged from 1.13% to 2.31% in the horse chestnut trees and from 1.52% to 2.86% in the lime-trees. The optimal potassium uptake positively affects water management in plants and it significantly increases their resistance to adverse climatic conditions (frost, drought) [31]. The potassium content in Nadolnik Park (0.78–3.34%) was generally higher than in our study, but the content measured in Wroclaw was much lower (on average 0.66%) [77].

In our study the calcium content in leaves ranged from 1.05% to 2.12% in the horse chestnut trees and from 1.43% to 2.04% in the lime-trees. These contents seem normal although the calcium content in the soil was considerably over the limit–there were no Ca: Mg or Ca: Fe antagonisms causing chlorosis [31]. Łukasiewicz [72] noted generally similar calcium content in horse chestnut leaves, i.e. 1.8% d. m. The calcium content measured in Wrocław was 5-fold lower (on average 0.38%) [77].

The content of magnesium in leaves ranged from 0.16% to 0.42% in the horse chestnut trees and from 0.19% to 0.49% in the lime-trees. Golcz et al. [49] observed similar contents of this nutrient. In our study the sulphur content ranged from 0.12% to 0.16% in horse chestnut leaves and from 0.17% to 0.32% in lime leaves. In general, the average sulphur content is species-dependent and ranges from 0.2% to 0.5% [31]. It affects plants’ resistance to frost and drought. Available reference publications do not provide comparative data on the content of sulphur in the leaves of horse chestnut and lime-trees.

According to Nikolic and Pavlovic [79], the optimal iron content in plants ranges from 100 to 300 mg kg^-1^ d. m., the manganese content–from 10–25 to 80 mg kg^-1^ d. m., and the copper content–from 2–5 to 20 mg kg^-1^ d. m. In general, the concentrations of these elements determined in our study were within these limits. The Mn content in leaves strongly depends on the soil pH. When it is elevated (it is characteristic of urban soils), the uptake of this microelement decreases [31].

The low sodium content in leaves was a positive aspect of our study. The sodium content in leaves in Wrocław was very high (on average 1.5% d.m.) [77]. The research conducted in Riga (Latvia) also showed high Na concentration in horse chestnut leaves (1.5%) and low Cl content. The excessive Na content was manifested by necrosis, which covered up to 80% of the leaf lamina [80]. The excessive Cl content in leaves might be the factor with the most negative effect on the health of trees in cities [77]. The research conducted on trees in Edmonton (Canada) also revealed that the increased sodium accumulation and the reduced chlorophyll content in the leaves were strongly correlated with the high soil salinity (EC-level) [81].

The accumulation of heavy metals in plants poses the risk of toxicity to humans and animals [82–84]. According to Kabata-Pendias [31], the maximum permissible content of cadmium in plants is 2 mg kg^-1^ d. m. The symptoms of cadmium toxicity may occur when the content of this metal exceeds 5 mg kg^-1^ d.m. The contents of heavy metals determined in the leaves were generally similar, except for two sites, i.e. T. Mazowiecki Park and Świętojański Park. It is most likely that this situation resulted from the proximity of a busy road, where lead contained in gasoline (tetraethyl lead) was emitted in the previous decade. The coefficients of variation referring to the content of cadmium and lead in leaves were low. They respectively amounted to 10.8% and 13.2% in the horse chestnut trees and to 13.6% and 12.7% in the lime-trees.

The contents of nutrients determined in the lime leaves in our study were similar to those quoted by Białobok [85], who used other authors’ research to give the contents of nutrients in the leaves of healthy small-leaved lime-trees. The author provided the following contents: 2.26%-2.50% N, 0.12%-0.35% P, 0.8%-1.5% K, 0.12%-0.38% Mg, 0.9%-2.5% Ca, 141–500 ppm Fe, 181–562 ppm Mn, 9–20 ppm Cu. The contents of nitrogen <1.9–2.0%, phosphorus <0.10% and potassium <0.80% in leaves should be regarded as deficient [85]. In our study the cadmium content ranged from 0.89 to 1.16 mg·kg^-1^, whereas the lead content ranged from 4.31 to 6.45 mg·kg^-1^. The range of the lead content was noticeably lower than the one Czarnowska [71] measured in small-leaved lime-trees growing in parks and along roads in Łódź (1.4–34.8 mg·kg^-1^), whereas the average cadmium content (0.10 mg·kg^-1^) was smaller than the content measured in our research.

The research conducted in Belgrade (Serbia) revealed significant accumulation of Cr, Fe, Ni, Zn and Pb in the leaves of *A*. *hippocastanum* L. and significant accumulation of Cr, Fe, Ni and Pb in the leaves of *Tilia* sp. This tendency was not observed for Cu [86]. The research conducted in Plovdiv (Bulgaria) showed that both the species and the site may significantly affect the content of heavy metals in plants. The zinc content tended to be more species dependent. The opposite dependency was observed for the Cr and Fe content and to some extent, for the Pb content in leaves, which mostly depended on the location of plants. The content of Cd and Cu did not depend significantly either on the species or location [32]. The available research results also point to the general principle that there was higher accumulation of heavy metals (Cr, Fe, Ni, Zn, Pb, As and V) in the leaves of *A*. *hippocastanum* L. trees [27,86,87]. *T*. *cordata* Mill. trees can be recommended as part of tall vegetation in housing estates, parks and other urbanised areas. They should not be grown along roads or in squares due to difficult environmental conditions and high pollution with Cu, Zn and Pb compounds, which are mostly emitted by various means of transport. *Tilia* trees can be grown more widely outside cities, especially in alleys along roads [88,89].

Earlier research showed that the mineral nutrition of trees growing in public green spaces may be imbalanced. There were lower concentrations of S, K and Mn in *Tilia* sp. and *A*. *hippocastanum* L. leaves [90]. Apart from that, increased soil contamination with Na and Cu could have negatively affected the development of mycorrhizal fungi in *Tilia* sp. [91]. The research conducted in Finland proved that both the type and age of a green area affected the soil pH, the total content of organic matter, the C and N concentration and microbiological processes [52]. It is hypothesised that as the content of organic matter in soil increases over time, it is more likely that carbon, nutrients and heavy metals may be immobilised [92]. Research showed that the total phosphorus content in soil was the smallest in the vicinity of dicotyledonous trees growing in new and middle-aged public green spaces. The phosphorus content was the highest in the oldest green areas. The same dependency was observed for heavy metals. The following correlations were also found: soil organic matter (SOM)–total P (r = 0.29, p < 0.001), SOM–Pb (r = 0.25, p < 0.001), SOM–Cu (r = 0.25, p < 0.001) and SOM–Zn (r = 0.17, p = 0.006). There was also a negative correlation between the soil pH and heavy metals. The soil pH and total P were not correlated [52].

### Health status of trees

It is very important to correctly select plant species for urban green spaces due to specific and difficult soil and climate characteristics [93]. Individual species of trees and shrubs exhibit different tolerance to unfavourable environmental conditions [46]. In general, horse chestnut trees grow well in cities [86]. Our study showed that the trees growing at the sites under analysis looked attractive, had compact habits, dark green leaves and showed no signs of diseases or nutrient deficiencies. According to Łukasiewicz and Oleksyn [94], the development of horse chestnut trees is negatively affected by anthropogenic changes such as asphalt or concrete surfaces near the tree trunk. The condition of the lime-trees was also satisfactory.

## Conclusions

The research led to the following conclusions:

1. In general, all the soil profiles were alkalised. It may have been caused by the elevated content of alkaline cations.
2. The soils under analysis were characterised by low salinity (EC), which ranged from 0.09 to 0.38 mS·cm^-1^ at the sites of the lime-trees and from 0.03 to 0.28 mS·cm^-1^ at the sites of the horse chestnut trees.
3. The coefficients of variation in the chemical composition of soils at the research sites were generally greater than the coefficients of variation in the chemical composition of leaves.
4. Due to the normal growth and appearance of the trees the following temporary guide values of nutrients in leaves can be proposed:
  - horse chestnut trees: (% d.m.): N 2.38%-4.71%, P 0.24%-0.46%, K 1.13%-2.31%, Ca 1.05%-2.12%, Mg 0.16%-0.42%, S 0.12%-0.23%; (mg kg^-1^ d.m.): Fe 89.8–198.8, Zn 17.6–33.1, Cu 7.36–19.61;
  - small-leaved lime-trees (% d. m.): N 2.45%-3.22%, P 0.27%-0.42%, K 1.52%-2.86%, Ca 1.43%-2.02%, Mg 0.19%-0.35%, S 0.19%-0.25%; (mg kg^-1^ d.m.): Fe 137.6–174.3, Zn 20.2–23.8, Cu 8.36–9.79 (mg kg^-1^ d. m.).
5. There was a significant negative dependency between the Mn content in the leaves and the health status of both species. This fact points to the need to conduct further research on the toxicity of this micronutrient to plants growing in urban areas.

This research was supported by the Ministry of Science and Higher Education of Poland as part of statutory activities.
